# Supplementary material for: Integrated Long-Term Care ‘Neighbourhoods’ to Support Older Populations: Evolving Strategies in Japan and England
Source: Int J Environ Res Public Health. 2023 Jul 12;20(14):6352. doi: 10.3390/ijerph20146352 (PMC10379849; doi:10.3390/ijerph20146352)
Supplement: Supplementary file 1 [file ijerph-20-06352-s001.zip › ijerph-2370311-supplementary.pdf]

Table S1. Development of Health and Welfare Policies for Older Adults in England

Date: Development	Major policies	
	Welfare/ Social Care	Healthcare
**1900s:** Early 20th Century Liberalism	1908 *Old Age Pensions Act*<br>1929 *Local Government Act*<br>◇ Poor Law power transfer to local authorities (LAs)s	1911 *National Health Insurance Act*<br>1929 Poor Law Hospitals become LA responsibility
**1940s:** Beginning of 'Welfare State'	1942 Beveridge: *Social Insurance & Allied Services*.<br>◇ Introduction of National Insurance	1944 White Paper, *A National Health Service*
**1946-48:** 'Welfare State' established (post World War II)	1948 *National Assistance Act* (Abolished Poor Law)<br>◇ Older people needing care & attention admitted to residential homes under LAs	*National Health Service (NHS) Bill*<br>◇ Hospitals incorporated into NHS.<br>◇ Older people who are sick admitted to hospital
**1970s:** NHS reorganization	1970 *Local Authority and Social Services Act*<br>◇ Established personal social services departments in local authorities (LAs)	1973 *NHS Reorganisation Act*<br>◇ Area health authorities (HAs) now responsible for services divided between hospital boards before
**1980s:** Supporting people 'in the community'	1981 White Paper, *Growing Older*<br>◇ Public services role 'to help people to care for themselves and their families'<br>1989 White Paper *Caring for People: Community care in the next decade and beyond*.	1980 *Health Services Act*<br>◇ Area HAs replaced by District HAs, many no longer coterminous with LAs<br>1987 White Paper, *Promoting Better Health*<br>◇ Improved patient choice
**1990s:** Funding of social care	1996 *Community Care (Direct Payments) Act*<br>◇ Direct payment to arrange own support for some<br>1998 White Paper, *Modernising Social Services: Promoting independence, improving protection, raising standards*<br>1999 Royal Commission on Long Term Care<br>◇ Ongoing funding of long-term care of older people	1990 *NHS and Community Care Act*<br>◇ NHS reorganization & Creation of internal market<br>1997 White Paper, *The new NHS: Modern, dependable*<br>1998 Acheson Inquiry - Inequalities in health

Table S1 (contd). Development of Health and Welfare Policies for Older Adults in England

Date: Development	Major policies	
	Welfare/ Social Care	Healthcare
**2000s:** Health & social care 'partnership'	2000 *Care Standards Act*<br>◇ Registration & regulation of care services, and social care workers<br>2001 *The National Service Framework for Older People*<br>◇ Standards to improve quality of support.	2000 *The NHS Plan*<br>◇ Development of intermediate care as bridge between hospital and home.<br>2006 White Paper, *Our Health, Our Care, Our Say*<br>◇ Reforms for people with long-term needs.
**2010s:** Beginning of 'integration' of health & care	2010 White Paper, *Building the National Care Service*<br>2011 Degree-level nurse education & specialist geriatric nurse training<br>2011 *Dilnot Review*<br>◇ Recommends major reforms to funding of adult social care	2010 *Health and Social Care Bill*<br>◇ Increased GP role commissioning health services<br>2019 *NHS Long Term Plan*<br>◇ Aims to improve patient care over next ten years
**2020s:**	2021 Post-pandemic recovery plan, *Build Back Better: Our Plan for Health and Social Care*<br>2021 White Paper *People at the Heart of Care: Adult Social Care Reform*<br>◇ Ten-year vision for adult social care reform	2021 White Paper, *Working together to improve health and social care for all*<br>2022 White Paper, *Joining up care for people, places and populations*<br>2022 *Health and Care Act*<br>◇ Legislation to integrate services for patients

Sources:

Wanless, D. (2006). Securing good care for older people: taking a long-term view. Retrieved from https://www.kingsfund.org.uk/sites/default/files/field/field_publication_file/securing-good-care-for-older-people-wanless-2006.pdf
Nuffield Trust. (2023). NHS reform timeline (1940s to 2020s). Retrieved from https://www.nuffieldtrust.org.uk/features/nhs-reform-timeline
